# Isolated Tuberculosis of Transplanted Liver, A Case Report and Review of the Literature

**DOI:** 10.5812/hepatmon.6691

**Published:** 2013-05-21

**Authors:** Bita Geramizadeh, Saman Nikeghbalian, Parisa Janghorban, Seyed Ali Malekhosseini

**Affiliations:** 1 Transplant Research Center, Shiraz University of Medical Science, Shiraz, IR Iran; 2 Department of Pathology, Shiraz University of Medical Science, Shiraz, IR Iran; 3 Transplant Ward, Shiraz University of Medical Science, Shiraz, IR Iran

**Keywords:** Liver Transplantation, Tuberculosis, Liver

## Abstract

**Introduction:**

Recipients of liver transplantation are prone to different types of infections such as tuberculosis (TB).

**Case Presentation:**

Herein we report a 59-year-old man with liver transplantation due to HBV cirrhosis who developed isolated hepatic TB, 18 months after OLT (orthotropic liver transplantation). He has been successfully treated with anti-TB regimen and now after 12 months he is completely symptom-free.

**Conclusions:**

Organ transplantation and treatment of transplanted patients with immunosuppressive drugs would prone them to various unusual infections. One of these is unusual primary involvement of liver by tuberculosis which has been extremely rare in the previous reports.

## 1. Introduction

Mycobacterium tuberculosis affects 0.47-2.3% of adults with liver transplantation (1) and the overall incidence is three times more than general population (2). Post-transplant TB is more frequent in Asia and Africa (3). It can be reactivation of a latent or primary post-transplant infection (4). Post-liver transplant TB can be either pulmonary or extra pulmonary such as lymph node, meninges, genitourinary and etc. (5). Post-liver transplant tuberculosis affecting transplanted liver has been rarely reported, most of which have been part of disseminated diseases. However, isolated hepatic tuberculosis in a transplanted liver is extremely rare and to the best of our knowledge three previous cases have been reported in the English literature (6-8). Herein we report our experience with such a case that has been successfully treated and also we will review the other previous cases of isolated hepatic allograft TB.

## 2. Case Presentation

A 59-year-old man underwent OLT in March 2010 for HBV cirrhosis. His pre-transplant work-up including PPD test (purified protein derivative test) and chest X-ray were negative. In his past medical history, he has been diabetic for the last 5 years which has been on insulin therapy. He received a liver from a cadaveric, blood group identical young man, victim of a car accident. According to his family, the cadaver had completely been healthy, with no prior disease. Patient’s post-transplant period was unremarkable and left the hospital in less than 10 days. During a year after transplant, he had no specific problem until August 2011, i.e. a month before admission when he developed anorexia, easy fatigability, malaise, night sweat and low grade fever. Physical examination showed: BP = 150/70, PR = 80/min, RR = 16/min, and T = 37.7 ⁰C. Other findings were mild icteric sclera and pale conjunctive.

Table 1 shows the laboratory examination findings: WBC= 6900/ml, and Hb = 9 gr/dl. Liver function tests (LFT) showed elevated ALT (60IU/L, normal < 40) and AST (55 IU/L, normal < 40). Alkaline phosphatase was also mildly elevated (385IU/L, normal 65-300).

**Table 1.** Summary of Laboratory Results of the Patient at the Time of Diagnosis of Liver Allograft Tuberculosis

Test	Results
**White blood cell count, WBC Count, ml**	6900
**Hemoglobin, gr/dl**	9
**Alanine Aminotransferase, IU/L (Normal < 40)**	60
**Aspartate Aminotransferase, IU/L(Normal < 40)**	55
**Alkaline Phosphatase, IU/L (Normal 65-300)**	385
**PCR-EBV**	Negative
**PCR-CMV**	Negative
**HBS Antibody titer, mIU/ml**	15
**HBV- viral load-PCR**	Zero

**Figure 1.**
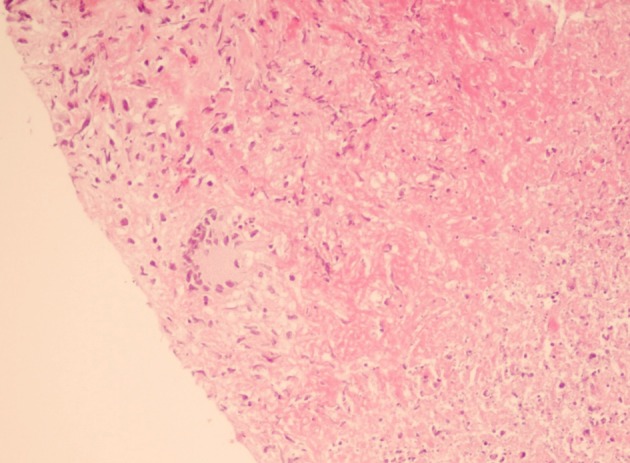
Histopathology Section From the Allograft Liver Shows Necrotizing Granuloma (H&EX250)

Quantitative PCR for EBV and CMV were negative. HBS-Ag was negative and HBS-Ab titer was 15 mIU/ml. HBV copy number was zero. At that time he was receiving Tacrolimus (1 mg/day, per oral (PO)), Sirolimus (2 mg/day, PO),Mycophenolate mophetil (1 gr/day, PO), Lamivudin (100 mg/day, PO) and HBV Immunoglobulin (HBIg) once/week intramuscularly (7). Liver biopsy was performed which showed caseating granuloma with positive acid fast bacilli (Figure 1). Chest and abdominal CT scan were unremarkable.

Diagnosis of TB was made and anti-TB therapy was started, composed of ethambutol 1200 mg/day, rifampicin 600 mg/day, and isoniazid (INH) 300 mg/day. Meanwhile tacrolimus was discontinued, and 5 mg/day (8) Methyl prednisolone was added to the drugs. After a short period sustained reduction of liver enzymes was noticed and also the patient’s general condition showed significant recovery. After 12 months of anti TB therapy he is completely well, laboratory findings are normal and drugs are gradually decreased to be followed for any further problem. He is still on the previous immunosuppressive regimen and there has been no episode of rejection during the last 12 months.

## 3. Discussion

Immunosuppressive therapy in transplanted recipients increases the incidence of tuberculosis, because of suppressive effects against the cell mediated immunity (9). Most cases of post-transplant tuberculosis in the literature have been among the renal transplant patients and reports of post liver transplant tuberculosis are not common (10). Isolated TB in the allograft liver is extremely rare and until now only three cases have been reported (6-8). Table 2 shows clinical characteristics of previous and current cases of isolated TB in the transplanted liver in detail.

**Table 2.** Clinical Characteristics of the Reported Cases of Isolated Hepatic TB After Liver Transplantation

Case	age	Sex	Immunosuppressives	post-transplant period, mo	Presenting symptoms	Underlying cause of cirrhosis
**Kiuchit et al (7)**	10 months	female	Tacrolimus/steroid	3	Fever	Biliary Atresia
**Alothman et al(6)**	43 years	male	Not known	18	High Fever	HCV
**Berzigotti et al (8)**	33 years	male	Tacrolimus/steroid	8	Fever	Cryptogenic
**Current case**	59 years	male	Tacrolimus,sirolimusMycophenolatemofetil Steroid	17	Fever ,night sweat	HBV

Two of the previous cases as well as our case have been in the patients above 40 years of age who have been transplanted by a cadaveric liver (6, 8). However, the patient reported by Kiuchit et al. From Japan has received the liver from her mother (who finally turned out to be PPD positive). In the adult cases of allograft TB, the diagnosis has been made in 8 to 18 months, but in the pediatric cases, this duration was much shorter i.e. 3 months post transplantation (6-8). All of the previous cases were PPD negative before transplantation, so it seems that they have got the infection from the graft (6-8). However, pre-transplant PPD test may underestimate the infection, because cirrhosis as chronic disease may result in cutaneous anergy which declines the sensitivity of the test, especially in our case with a history of diabetes mellitus (3). Addition of pre-transplantion chest imaging would be helpful (1). It is also important to perform PPD test and chest X-ray in the donor if it’s feasible (living related transplant) (3). Most common presenting symptoms have been fever and night sweat which in combination with abnormal LFT has led to perform a liver biopsy (7, 8). The intensity of immunosuppression is a possible risk factor for the development of post-transplant TB i.e. OKT3 or anti-T lymphocyte antibodies (4). However none of the previous post-liver transplant tuberculosis cases has been receiving these types of medication. Presence of other accompanying diseases such as diabetes (as in our case) is also another predisposing factor (4). Tissue biopsy is critical for the establishment of the diagnosis (1). All the previous cases have been diagnosed by liver biopsy or fine needle aspiration and the presence of acid fast bacilli and caseating granuloma (6-8). Treatment of the hepatic TB in the allografts of previous cases has been successful by anti TB regimens composed of INH, refampin, and ethambutol. In none of them INH hepatotoxicity has been reported despite of at least 6 months of therapy (6-8). It is very important to monitor the patients with liver transplant on both anti TB and immunosuppressive drugs, in order to prevent graft rejection and maintain an effective anti TB therapy, because of high mortality of untreated TB in a transplant recipient (11). As a conclusion high degree of suspicion is necessary for diagnosis of post-liver hepatic transplant TB and should be considered in the patients with fever and night sweat as well as abnormal LFT.
